# Existence of weakly quasisymmetric magnetic fields without rotational transform in asymmetric toroidal domains

**DOI:** 10.1038/s41598-022-15594-9

**Published:** 2022-07-05

**Authors:** Naoki Sato

**Affiliations:** Graduate School of Frontier Sciences, The University of Tokyo, Kashiwa, Chiba 277-8561 Japan

**Keywords:** Magnetically confined plasmas, Pure mathematics

## Abstract

A quasisymmetry is a special symmetry that enhances the ability of a magnetic field to trap charged particles. Quasisymmetric magnetic fields may allow the realization of next generation fusion reactors (stellarators) with superior performance when compared with tokamak designs. Nevertheless, the existence of such magnetic configurations lacks mathematical proof due to the complexity of the governing equations. Here, we prove the existence of weakly quasisymmetric magnetic fields by constructing explicit examples. This result is achieved by a tailored parametrization of both magnetic field and hosting toroidal domain, which are optimized to fulfill quasisymmetry. The obtained solutions hold in a toroidal volume, are smooth, possess nested flux surfaces, are not invariant under continuous Euclidean isometries, have a non-vanishing current, exhibit a vanishing rotational transform, and fit within the framework of anisotropic magnetohydrodynamics. Due to the vanishing rotational transform, these solutions are however not suitable for particle confinement.

## Introduction

**Figure 1:**
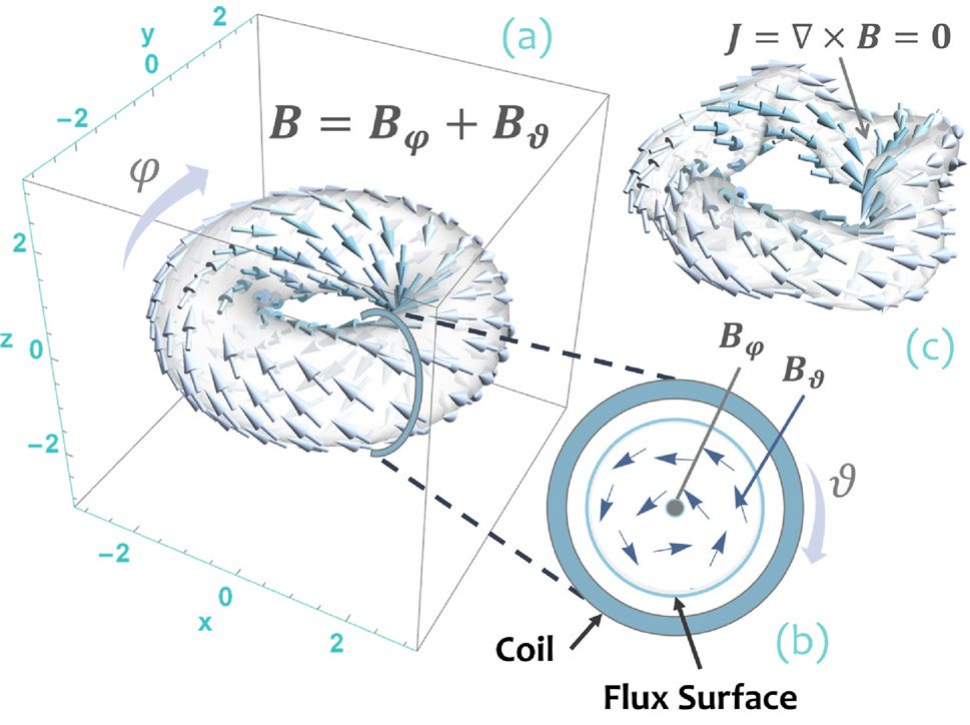
(**a**) and (**b**): magnetic field configuration in an axially symmetric tokamak. The total confining magnetic field $\varvec{B}=\varvec{B}_{\varphi }+\varvec{B}_{\vartheta }$ is given by an axial (toroidal) component $\varvec{B}_{\varphi }$ produced by external coils plus a poloidal component $\varvec{B}_{\vartheta }$ generated by an electric current flowing in the $\varphi$-direction. This current is sustained by the confined plasma itself. Here, $\varphi$ and $\vartheta$ denote toroidal angle and poloidal angle respectively. For simplicity, the reactor vessel separating external coils from the confinement region is not shown. (**a**) The total magnetic field $\varvec{B}$ over a flux surface $\Psi =\mathrm{constant}$ such that $\varvec{B}\cdot \nabla \Psi =0$. (**b**) Schematic view of toroidal component $\varvec{B}_{\varphi }$ and poloidal component $\varvec{B}_{\vartheta }$ on a cross section $\varphi =\mathrm{constant}$. (**c**) Schematic representation of a stellarator: the confining magnetic field is asymmetric and entirely produced by external coils, implying that the associated electric current vanishes in the confinement region, $\varvec{J}=\nabla \times \varvec{B}=\varvec{0}$. Figure created using Wolfram Mathematica 12.2 (www.wolfram.com/mathematica).

Nuclear fusion is a technology with the potential to revolutionize the way energy is harvested. In the approach to nuclear fusion based on magnetic confinement, charged particles (the plasma fuel) are trapped in a doughnut-shaped (toroidal) reactor with the aid of a suitably designed magnetic field. In a tokamak^1^, the reactor vessel is axially symmetric (see Fig. 1a). The axial symmetry is mathematically described by the independence of physical quantities, such as the magnetic field $\varvec{B}$ and its modulus *B*, from the toroidal angle $\varphi$. Such symmetry is crucial to the quality of tokamak confinement, because it ensures the conservation of the angular momentum $p_{\varphi }$ of charged particles. However, the constancy of $p_{\varphi }$ is not enough to constrain particle orbits in a limited volume because, in addition to the tendency to follow magnetic field lines, particles drift across the magnetic field. This perpendicular drift eventually causes particle loss at the reactor wall, deteriorating the confinement needed to sustain fusion reactions. In a tokamak, perpendicular drifts are therefore suppressed by driving an axial electric current through the confinement region, which generates a poloidal magnetic field in addition to the external magnetic field produced by coils surrounding the confinement vessel (see Figs. 1a, b). The overall magnetic field therefore forms twisted helical field lines around the torus. Unfortunately, the control of such electric current is difficult because it is maintained by the circulation of the burning fuel itself, making steady operation of the machine a practical challenge.

In contrast to tokamaks, stellarators^2,3^ are designed to confine charged particles through a vacuum magnetic field produced by suitably crafted asymmetric coils (see Fig. 1c). In this context, symmetry is defined as invariance under continuous Euclidean isometries, i.e. transformations of three-dimensional Euclidean space that preserve the Euclidean distance between points. In practice, these transformations are combinations of translations and rotations, with three corresponding types of symmetry: translational, rotational (including axial), and helical. The magnetic field generated by the asymmetric coils of a stellarator is endowed with the field line twist required to minimize particle loss associated with perpendicular drift motion. This removes, in principle, the need to drive an electric current within the confinement region, and thus enables the reactor to operate in a condition close to a steady state (in practice currents may exist in stellarators as well, but they are sensibly smaller than those in a tokamak). Unfortunately, the loss of axial symmetry comes at a heavy price: in general, the angular momentum $p_{\varphi }$ is no longer constant, and confinement is degraded. However, a conserved momentum that spatially constrains particle orbits can be restored if the magnetic field satisfies a more general kind of symmetry, the so-called quasisymmetry^3,4^. The essential feature of a quasisymmetric magnetic field, whose rigorous definition^5^ is given in Eq. (1), is the invariance $\varvec{u}\cdot \nabla B=0$ of the modulus $B=\left|{\varvec{B}}\right|$ in a certain direction in space $\varvec{u}$ (the quasisymmetry). For completeness, it should be noted that there exist two kinds of quasisymmetry^6–9^: weak quasisymmetry (the one considered in the present paper), and strong quasisymmetry. In the former, quasisymmetry results in a conserved momentum at first order in the guiding-center expansion, while in the latter the conservation law originates from an exact symmetry of the guiding-center Hamiltonian. Furthermore, the notion of quasisymmetry can be generalized to omnigenity, a property that guarantees the suppression of perpendicular drifts on average^10^.

Despite the fact that several stellarators aiming at quasisymmetry or omnigenity have been built^11,12^, that significant efforts are being devoted to stellarator optimization (see e.g.^13^), and that quasisymmetric magnetic fields have been obtained with high numerical accuracy^14^, at present the existence of quasisymmetric magnetic fields lacks mathematical proof. This deficiency is rooted in the complexity of the partial differential equations governing quasisymmetry, which are among the hardest in mathematical physics. Indeed, on one hand the toroidal volume where the solution is sought is itself a variable of the problem. On the other hand, since the governing equations belong to the class of first order partial differential equations, it is difficult to establish general results beyond the existence of local solutions by application of standard analytical tools such as the method of characteristics. The availability of quasisymmetric magnetic fields also strongly depends on the additional constraints that are imposed on the magnetic field. For example, if a quasisymmetric magnetic field is sought within the framework of ideal isotropic magnetohydrodynamics, the analysis of^15^ suggests that such configurations do not exist (see also^16–19^) due to an overdetermined system of equations where geometrical constraints outnumber the available degrees of freedom. The issue of overdetermination is less severe^20–22^ if quasisymmetric mgnetic fields correspond to equilibria of ideal anisotropic magnetohydrodynamics^23–25^ where scalar pressure is replaced by a pressure tensor. In this context, it has been shown^26^ that local quasisymmetric magnetic fields do exist, although such local solutions are only defined in a portion of a toroidal domain due to a lack of periodicity around the torus.

The goal of the present paper is to establish the existence of weakly quasisymmetric magnetic fields in toroidal domains by constructing explicit examples. This ‘constructive’ approach has the advantage of bypassing the intrinsic difficulty of the general equations governing quasisymmetry, and hinges upon the method of Clebsch parametrization^27^, which provides an effective representation of the involved variables, including the shape of the boundary enclosing the confinement region. The quasisymmetric magnetic fields reported in the present paper hold within asymmetric toroidal volumes, are smooth, have nested flux surfaces, are not invariant under continuous Euclidean isometries, and can be regarded as quilibria of ideal anisotropic magnetohydrodynamics. Nevertheless, these results come with some caveats: since the constructed solutions are optimized only to fulfill weak quasisymmetry, the found magnetic fields lack other features that would be desirable from a confinement perspective. In particular, they exhibit a vanishing rotational transform (the number of poloidal transits made by a magnetic field line during a toroidal transit is zero), they are not vacuum fields, and their quasisymmetry does not lie on toroidal flux surfaces. Hence, despite being quasisymmetric, the constructed solutions are not suitable to confine particles within a bounded region. Whether additional properties such as a non-vanishing rotational transform or a vanishing current are consistent with weak quasisymmetry therefore remains an open theoretical issue.

## Construction of quasisymmetric magnetic fields

Let $\Omega \subset \mathbb {R}^3$ denote a smooth bounded domain with boundary $\partial \Omega$. In the context of stellarator design $\Omega$ represents the volume occupied by the magnetically confined plasma, while the bounding surface $\partial \Omega \simeq \mathrm{T}^2$ has the topology of a torus (a 2-dimensional manifold of genus 1). It is important to observe that, in contrast with conventional tokamak design, the vessel $\partial \Omega$ of a stellarator does not exhibit neither axial nor helical symmetry. In $\Omega$, a stationary magnetic field $\varvec{B}\left( {\varvec{x}}\right)$ is said to be weakly quasisymmetric provided that there exist a vector field $\varvec{u}\left( {\varvec{x}}\right)$ and a function $\zeta \left( {\varvec{x}}\right)$ such that the following system of partial differential equations holds,

1a $$\begin{aligned} \nabla \cdot \varvec{B}&=0,~~~~\varvec{B}\times \varvec{u}=\nabla \zeta ,~~~~\nabla \cdot \varvec{u}=0,~~~~\varvec{u}\cdot \nabla B^2=0~~~~\mathrm{in}~~\Omega , \end{aligned}$$

1b $$\begin{aligned} \varvec{B}\cdot \varvec{n}&=0~~~~\mathrm{on}~~\partial \Omega , \end{aligned}$$

where $B=\left|{\varvec{B}}\right|$ is the modulus of $\varvec{B}$, $\varvec{n}$ denotes the unit outward normal to $\partial \Omega$, and $\varvec{u}$ is the direction of quasisymmetry. As previously explained, system (1a) ensures the existence of a conserved momentum at first order in the guiding center ordering that is expected to improve particle confinement. Usually, the function $\zeta$ is identified with a flux function $\Psi$ having toroidal level sets. Then, both $\varvec{B}$ and $\varvec{u}$ lie on toroidal flux surfaces $\Psi =\mathrm{constant}$ and the conserved momentum originating from the quasisymmetry is well approximated by the flux function $\Psi$. Although this property is highly desirable from a confinement perspective because it confines particle orbits into a bounded region, in principle weak quasisymmetry (1) can be fulfilled even if the level sets of $\zeta$ differ from toroidal surfaces (see e.g.^5^). In particular, allowing configurations with $\zeta \ne \Psi$ leaves the interesting possibility of achieving good confinement if the level sets of $\zeta$ enclose bounded regions with a topology that may depart from a torus. Mathematically, the four equations in system (1a) represent so-called Lie-symmetries of the solution, i.e. the vanishing of the Lie-derivative $\mathfrak {L}_{\varvec{\xi }}T$ quantifying the infinitesimal difference between the value of a tensor field *T* at a given point and that obtained by advecting the tensor field along the flow generated by the vector field $\varvec{\xi }$. Specifically, the first equation and the third equation, which imply that both $\varvec{B}$ and $\varvec{u}$ are solenoidal vector fields, express conservation of volumes advected along $\varvec{B}$ and $\varvec{u}$ according to $\mathfrak {L}_{\varvec{B}}dV=\mathfrak {L}_{\varvec{u}}dV=\left( {\nabla \cdot \varvec{B}}\right) dV=\left( {\nabla \cdot \varvec{u}}\right) dV=0$, where $dV=dxdydz$ is the volume element in $\mathbb {R}^3$. Similarly, the second equation in (1a) expresses the invariance of the vector field $\varvec{B}$ along $\varvec{u}$ according to $\mathfrak {L}_{\varvec{u}}\varvec{B}=\varvec{u}\cdot \nabla \varvec{B}-\varvec{B}\cdot \nabla \varvec{u}=\nabla \times \left( {\varvec{B}\times \varvec{u}}\right) =\varvec{0}$, while the fourth equation expresses the invariance of the modulus $B^2$ along $\varvec{u}$, i.e. $\mathfrak {L}_{\varvec{u}}B^2=\varvec{u}\cdot \nabla B^2=0$. For further details on these points see^26^.

The construction of a solution of (1) is complicated by the fact that $\varvec{B}$, $\varvec{u}$, $\zeta$ and $\partial \Omega$ are not independent parameters, but they must be optimized in a concurrent fashion while respecting the topological requirements on the shape of the bounding surface. For example, assigning the bounding surface $\partial \Omega$ from the outset will generally prevent the existence of solutions due to overdetermination (the available degrees of freedom are not sufficient to satisfy the quasisymmetry equations). A convenient way to simultaneously optimize $\varvec{B}$, $\varvec{u}$, $\zeta$, and $\partial \Omega$ is to use Clebsch parameters^27^, which enable the enforcement of the topological requirement on $\partial \Omega$, which must be a torus, and the extraction of the remaining geometrical degrees of freedom from $\varvec{B}$, $\varvec{u}$, and $\zeta$. To see this, first observe that the boundary $\partial \Omega$ can be expressed as a level set of a flux function $\Psi$ (which is assumed to exist) such that $\varvec{B}\cdot \nabla \Psi =0$ in $\Omega$. In particular, this implies that the unit outward normal to the boundary $\partial \Omega$ can be written as $\varvec{n}=\nabla \Psi /\left|{\nabla \Psi }\right|$. Next, parametrize $\varvec{B}$ and $\varvec{u}$ as

2 $$\begin{aligned} \varvec{B}=\nabla \beta _1\times \nabla \beta _2 ,~~~~\varvec{u}=\nabla u_1\times \nabla u_2, \end{aligned}$$

where the Clebsch parameters $\beta _1$, $\beta _2$, $u_1$, and $u_2$ are (possibly multivalued) functions that must be determined from the quasisymmetry Eqs. (1) and the topological requirement that $\Psi$ defines toroidal surfaces. Here, it should be noted that, due to the Lie-Darboux theorem^28^, for a given smooth solenoidal vector field $\varvec{v}$ one can always find single valued functions $\alpha _1$ and $\alpha _2$ defined in a sufficiently small neighborhood *U* of a chosen point $\varvec{x}\in \Omega$ such that $\varvec{v}=\nabla \alpha _1\times \nabla \alpha _2$ in *U*. In light of the parametrization (2), the boundary condition $\varvec{B}\cdot \varvec{n}=\varvec{B}\cdot \frac{\nabla \Psi }{\left|{\nabla \Psi }\right|}=0$ on $\partial \Omega$ can now be identically satisfied by demanding that $\Psi =\Psi \left( {\beta _1,\beta _2}\right)$. Furthermore, assuming $\varvec{u}\ne \varvec{0}$, the fourth equation in (1a) implies that the modulus $B^2$ must be a function $f_B\left( {u_1,u_2}\right)$ of $u_1$ and $u_2$. Thus, using the parametrization (2), system (1) reduces to

3 $$\begin{aligned} \left( {\nabla \beta _1\times \nabla \beta _2}\right) \times \left( {\nabla u_1\times \nabla u_2}\right) =\nabla \zeta ,~~~~\left|{\nabla \beta _1\times \nabla \beta _2}\right|^2=f_B\left( {u_1,u_2}\right) ,~~~~\Psi =\Psi \left( {\beta _1,\beta _2}\right) . \end{aligned}$$

In going from (1)–(3) we used the fact that the first and third equations in (1a) are identically satisfied.

Now our task is to solve system (3) by determining $\beta _1$, $\beta _2$, $u_1$, $u_2$, $f_B$, $\zeta$, and $\Psi$ so that the level sets of $\Psi$ define toroidal surfaces. Direct integration of (3) is a mathematically difficult task due to the number and complexity of the geometric constraints involved. Therefore, it is convenient to start from known special solutions corresponding to axially symmetric configurations, and then perform a tailored symmetry breaking generalization. The simplest axially symmetric vacuum magnetic field is given by

4 $$\begin{aligned} \varvec{B}_0=\nabla \varphi =\nabla z\times \nabla \log r. \end{aligned}$$

The magnetic field (4) satisfies system (1) if, for example, the quasisymmetry is chosen as $\varvec{u}_0=\varvec{B}_0$. The corresponding flux surfaces are given by axially symmetric tori generated by level sets of the function

5 $$\begin{aligned} \Psi _0=\frac{1}{2}\left[ \left( {r-r_0}\right) ^2+z^2\right] , \end{aligned}$$

with $r_0$ a positive real constant representing the radial position of the toroidal axis (major radius). Comparing Eq. (2) with Eqs. (4) and (5), one sees that $\beta _1=u_1=z$, $\beta _2=u_2=\log r$, $B_0^2=1/r^2=e^{-2u_2}$, and $\Psi _0=\frac{1}{2}\left[ \left( {e^{\beta _2}-r_0}\right) ^2+\beta _1^2\right]$.

The axially symmetric torus (5) can be generalized to a larger class of toroidal surfaces^26^ as

6 $$\begin{aligned} \Psi =\frac{1}{2}\left[ \left( {\mu -\mu _0}\right) ^2+\mathcal {E}\left( {z-h}\right) ^2\right] . \end{aligned}$$

**Figure 2:**
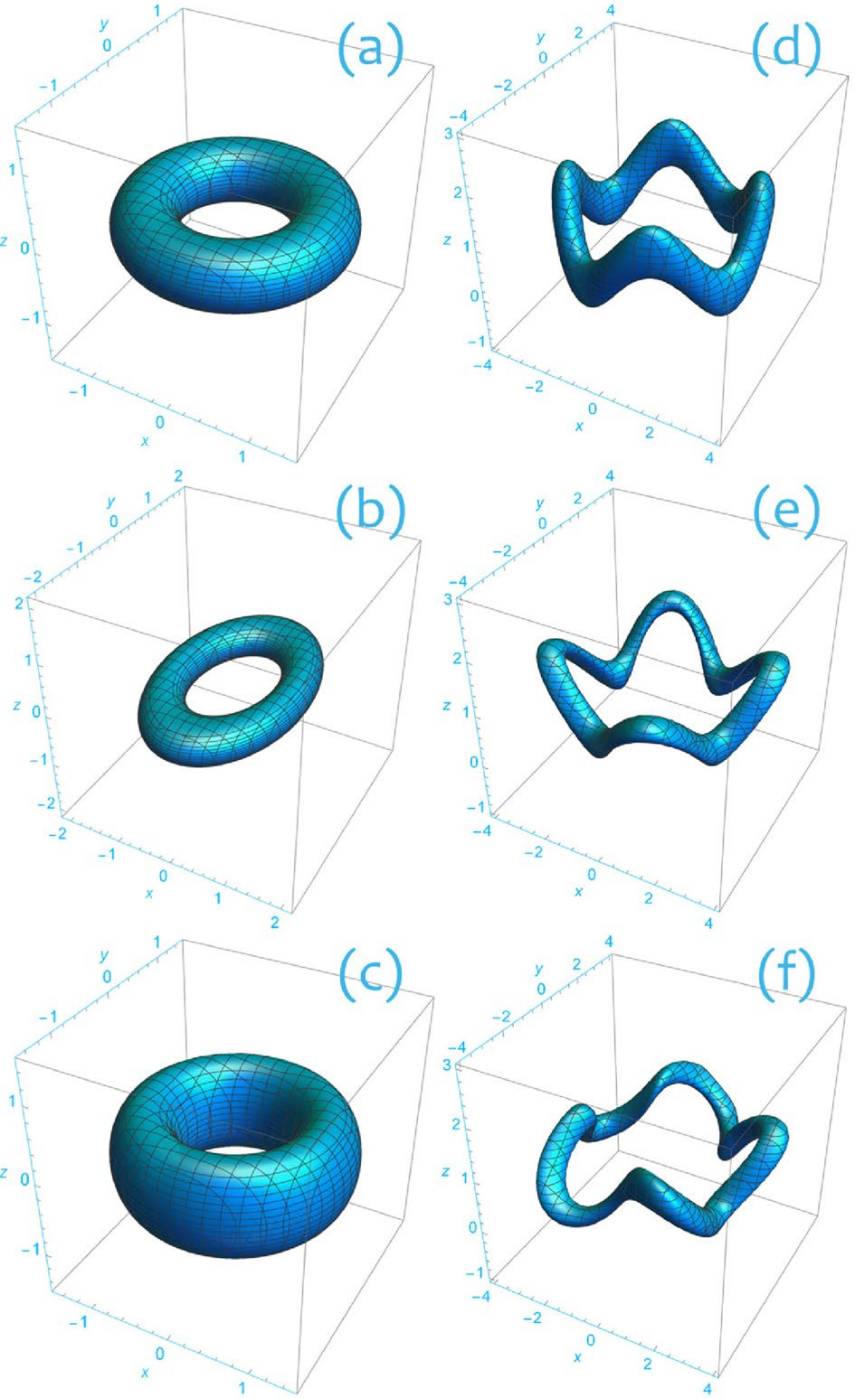
Toroidal surfaces obtained as level sets of the function $\Psi$ defined by Eq. (6). (**a**) Axially symmetric torus $\Psi =0.15$ with $\mu =r$, $\mu _0=1$, $\mathcal {E}=1$, and $h=0$. (**b**) Elliptic torus $\Psi =0.1$ with $\mu =\sqrt{x^2+0.4 y^2}$, $\mu _0=1$, $\mathcal {E}=1$, and $h=0$. Notice that sections $z=\mathrm{constant}$ form ellipses. (**c**) Axially symmetric torus $\Psi =0.15$ with $\mu =r$, $\mu _0=1$, $\mathcal {E}=0.4$, and $h=0$. Notice that sections $\varphi =\mathrm{constant}$ form ellipses. (**d**) Torus $\Psi =0.1$ with $\mu =r$, $\mu _0=3$, $\mathcal {E}=1$, and $h=1+0.5\sin \left( {4\varphi }\right)$. (**e**) Torus $\Psi =0.1$ with $\mu =r$, $\mu _0=3+0.5\sin \left( {4\varphi }\right)$, $\mathcal {E}=5+2.5\sin \left( {4\varphi }\right)$, and $h=1+0.5\sin \left( {4\varphi }\right)$. (**f**) Torus $\Psi =0.1$ with $\mu =\sqrt{x^2+(0.9+0.1\sin \left( {3\varphi }\right) )y^2}$, $\mu _0=3+0.5\sin \left( {5\varphi }\right)$, $\mathcal {E}=5+2.5\cos \left( {3\varphi }\right)$, and $h=1+0.5\sin \left( {4\varphi }\right)$. Figure created using Wolfram Mathematica 12.2 (www.wolfram.com/mathematica).

In this notation, $\mu$, $\mu _0$, $\mathcal {E}$, and *h* are single valued functions with the following properties. For each *z*, the function $\mu$ measures the distance of a point in the $\left( {x,y}\right)$ plane from the origin in $\mathbb {R}^2$. The simplest of such measures is the radial coordinate *r*. More generally, on each plane $z=\mathrm{constant}$ level sets of $\mu$ may depart from circles and exhibit, for example, elliptical shape. The function $\mu _0$ assigns the $\mu$ value at which the toroidal axis is located. For the axially symmetric torus $\Psi _0$, we have $\mu _0=r_0$. The function $\mathcal {E}>0$ expresses the departure of toroidal cross sections (intersections of the torus with level sets of the toroidal angle) from circles. For example, the axially symmetric torus $\Psi _\mathrm{ell}=\frac{1}{2}\left[ \left( {r-r_0}\right) ^2+2z^2\right]$ corresponding to $\mathcal {E}=2$ has elliptic cross section. Finally, the function *h* can be interpreted as a measure of the vertical displacement of the toroidal axis from the $\left( {x,y}\right)$ plane. Figure 2 shows different toroidal surfaces generated through (6).

The axial symmetry of the torus $\Psi _0$ given by (5) can be broken by introducing dependence on the toroidal angle $\varphi$ in one of the functions $\mu$, $\mu _0$, $\mathcal {E}$, or *h* appearing in (6). Let us set $\mu =r$, take $\mu _0$ and $\mathcal {E}$ as positive constants, and consider a symmetry breaking vertical axial displacement $h=h\left( {r,\varphi ,z}\right)$. For the corresponding $\Psi$ to define a toroidal surface, the function *h* must be single valued. Hence, $\varphi$ must appear in *h* as the argument of a periodic function. The simplest ansatz for *h* is therefore

7 $$\begin{aligned} h=\epsilon \sin \left[ m\varphi +g\left( {r,z}\right) \right] . \end{aligned}$$

Here $m\in \mathbb {Z}$ is an integer, $\epsilon$ a positive control parameter such that the standard axially symmetric magnetic field $\varvec{B}_0$ with flux surfaces $\Psi _0$ can be recovered in the limit $\epsilon \rightarrow 0$, and *g* a function of *r* and *z* to be determined. Now recall that from Eq. (3) the function $\Psi$ is related to the Clebsch potentials $\beta _1$ and $\beta _2$ generating the magnetic field $\varvec{B}=\nabla \beta _1\times \nabla \beta _2$ according to $\Psi \left( {\beta _1,\beta _2}\right)$. Comparing with the axially symmetric case (5) we therefore deduce that the analogy holds if $\beta _1=z-h$ and $\beta _2=\log r$. Defining $\eta =m\varphi +g$, it follows that the candidate quasisymmetric magnetic field is

8 $$\begin{aligned} \varvec{B}=\nabla \left( z-\epsilon \sin \eta \right) \times \nabla \log r=\left( 1-\epsilon \cos \eta \frac{\partial g}{\partial z}\right) \nabla \varphi +\epsilon m\frac{\cos \eta }{r^2}\nabla z, \end{aligned}$$

where *g* must be determined by enforcing quasisymmetry. Next, observe that

9 $$\begin{aligned} B^2=\frac{1}{r^2}\left[ \epsilon ^2m^2\frac{\cos ^2\eta }{r^2}+\left( {1-\epsilon \cos \eta \frac{\partial g}{\partial z}}\right) ^2\right] . \end{aligned}$$

An essential feature of quasisymmetry (3) is that the modulus $B^2$ can be written as a function of two variables only, $B^2=f_B\left( {u_1,u_2}\right)$. From Eq. (9) one sees that this result can be achieved by setting $\partial g/\partial z=q\left( {r}\right)$ for some radial function $q\left( {r}\right)$ so that $u_1=\eta$, $u_2=\log r$, and also

10 $$\begin{aligned} g\left( {r,z}\right) =q\left( {r}\right) z+v\left( {r}\right) , \end{aligned}$$

with $v\left( {r}\right)$ a radial function. The candidate direction of quasisymmetry is therefore

11 $$\begin{aligned} \varvec{u}=\sigma \left( {\eta ,r}\right) \nabla \eta \times \nabla \log r=\sigma \left( {\eta ,r}\right) \left( {q\nabla \varphi -\frac{m}{r^2}\nabla z}\right) , \end{aligned}$$

with $\sigma \left( {\eta ,r}\right)$ a function of $\eta$ and *r* to be determined. Since by construction $B^2=B^2\left( {u_1,u_2}\right)$, $\Psi =\Psi \left( {\beta _1,\beta _2}\right)$, and both $\varvec{B}$ and $\varvec{u}$ as given by (8) and (11) are solenoidal, the only remaining equation in system (3) to be satisfied is the first one. In particular, we have

12 $$\begin{aligned} \varvec{B}\times \varvec{u}=\sigma \left( {\nabla \varphi -\epsilon \cos \eta \nabla \eta \times \nabla \log r}\right) \times \left( {\nabla \eta \times \nabla \log r}\right) =-m\frac{\sigma }{r^3}\nabla r. \end{aligned}$$

Hence, upon setting $\sigma =\sigma \left( {r}\right)$, system (3) is satisfied with

13 $$\begin{aligned} \zeta =-m\int \frac{\sigma }{r^3}dr. \end{aligned}$$

Without loss of generality, we may set $\sigma =-r^3$ so that $\zeta =mr$ and the quasisymmetric configuration is given by

14a $$\begin{aligned} \varvec{B}=&\nabla \left[ z-\epsilon \sin \left( {m\varphi +qz+v}\right) \right] \times \nabla \log r=\left[ 1-\epsilon \cos \left( {m\varphi +qz+v}\right) q\right] \nabla \varphi +\epsilon m\frac{\cos \left( {m\varphi +qz+v}\right) }{r^2}\nabla z, \end{aligned}$$

14b $$\begin{aligned} \varvec{u}=&-\frac{1}{3}\nabla \left( {m\varphi +qz}\right) \times \nabla r^3=m r\nabla z-qr^3\nabla \varphi ,\end{aligned}$$

14c $$\begin{aligned} \Psi =&\frac{1}{2}\left\{ \left( {r-r_0}\right) ^2+\mathcal {E}\left[ z-\epsilon \sin \left( {m\varphi +qz+v}\right) \right] ^2\right\} , \end{aligned}$$

where $\mathcal {E}$ is a positive real constant.

## Verification of asymmetry

For the family of solutions (14) to qualify both as quasisymmetric and without continuous Euclidean isometries, we must verify that the magnetic field (14a) is not invariant under some appropriate combination of translations and rotations. To see this, consider the case $q=1/r$ and $v=0$ corresponding to

15a $$\begin{aligned} \varvec{B}=&\nabla \left[ z-\epsilon \sin \left( {m\varphi +\frac{z}{r}}\right) \right] \times \nabla \log r=\left[ 1-\epsilon \frac{\cos \left( {m\varphi +\frac{z}{r}}\right) }{r}\right] \nabla \varphi +\epsilon m\frac{\cos \left( {m\varphi +\frac{z}{r}}\right) }{r^2}\nabla z, \end{aligned}$$

15b $$\begin{aligned} \varvec{u}=&-\frac{1}{3}\nabla \left( {m\varphi +\frac{z}{r}}\right) \times \nabla {r^3}=m r\nabla z-r^2\nabla \varphi ,\end{aligned}$$

15c $$\begin{aligned} \Psi =&\frac{1}{2}\left\{ \left( {r-r_0}\right) ^2+\mathcal {E}\left[ z-\epsilon \sin \left( {m\varphi +\frac{z}{r}}\right) \right] ^2\right\} , \end{aligned}$$

where $\mathcal {E}$ is a positive real constant. Notice that the magnetic field (15a) is smooth in any domain $V\subset \mathbb {R}^3$ not containing the vertical axis $r=0$. To exclude the existence of any continuous Euclidean isometry for (15a) it is sufficient to show that the equation

16 $$\begin{aligned} \mathfrak {L}_{\varvec{\xi }}B^2=\varvec{\xi }\cdot \nabla B^2=0,~~~~\varvec{\xi }=\varvec{a}+\varvec{b}\times \varvec{x}, \end{aligned}$$

does not have solution for any choice of constant vector fields $\varvec{a},\varvec{b}\in \mathbb {R}^3$ with $\varvec{a}^2+\varvec{b}^2\ne 0$. Indeed, since $\varvec{\xi }=\varvec{a}+\varvec{b}\times \varvec{x}$ represents the generator of continous Euclidean isometries, the impossibility of satisfying (16) prevents the magnetic field $\varvec{B}$ from possessing translational, axial, or helical symmetry. For further details on this point, see^26^. Next, introducing again $\eta =m\varphi +z/r$, from Eq. (15a) one has

17 $$\begin{aligned} B^2=\frac{1}{r^2}-2\epsilon \frac{\cos \eta }{r^3}+\epsilon ^2\left( {1+m^2}\right) \frac{\cos ^2\eta }{r^4}. \end{aligned}$$

It follows that

18 $$\begin{aligned} \begin{aligned} \varvec{\xi }\cdot \nabla B^2=&\frac{2}{r^3}\left[ -1+3\epsilon \frac{\cos \eta }{r}-2\epsilon ^2\left( {1+m^2}\right) \frac{\cos ^2\eta }{r^2} \right] \varvec{\xi }\cdot \nabla r\\ {}&+2\epsilon \frac{\sin \eta }{r^3}\left[ 1-\epsilon \left( {1+m^2}\right) \frac{\cos \eta }{r}\right] \varvec{\xi }\cdot \nabla \eta . \end{aligned} \end{aligned}$$

Let $\left( {a_x,a_y,a_z}\right)$ and $\left( {b_x,b_y,b_z}\right)$ denote the Cartesian components of $\varvec{a}$ and $\varvec{b}$. On the surface $\eta =0$, corresponding to $z=z\left( {x,y}\right) =-mr\varphi =-m\arctan \left( {y/x}\right) \sqrt{x^2+y^2}$, we have $\sin \eta =0$ and $\cos \eta =1$, and therefore,

19 $$\begin{aligned} \varvec{\xi }\cdot \nabla B^2=\frac{2}{r^4}\left[ -1+\frac{3\epsilon }{r}-2\epsilon ^2\frac{\left( {1+m^2}\right) }{r^2}\right] \left[ xa_x+ya_y+\left( {xb_y-yb_x}\right) z\left( {x,y}\right) \right] . \end{aligned}$$

This quantity vanishes provided that $a_x=a_y=b_x=b_y=0$. Consider now the surface $\eta =\pi /2$, which implies $z=z\left( {x,y}\right) =r\left( {\pi /2-m\varphi }\right) =\sqrt{x^2+y^2}\left( {\pi /2-m\arctan \left( {y/x}\right) }\right)$. In this case $\sin \eta =1$ while $\cos \eta =0$. Furthermore, since the only surviving components in $\varvec{\xi }$ are those coming from $a_z$ and $b_z$, one has $\varvec{\xi }\cdot \nabla r=0$, and therefore

20 $$\begin{aligned} \varvec{\xi }\cdot \nabla B^2=\frac{2\epsilon }{r^3}\left( {\frac{a_z}{r}+mb_z}\right) . \end{aligned}$$

This quantity vanishes provided that $a_z=b_z=0$. Hence, the quasisymmetric magnetic field (15a) cannot possess continuous Euclidean isometries. Equation (20) also suggests that the magnetic field (15a) is endowed with a generalized kind of helical symmetry (although this symmetry does not correspond to an isometry of $\mathbb {R}^3$). Indeed, in a helically symmetric magnetic field one expects $B^2=B^2\left( {r,m\varphi +z}\right)$ for some constant *m*. However, the obtained solution (15a) is such that $B^2=B^2\left( {r,m\varphi +z/r}\right)$ as clear from (17). In this sense, the magnetic field (15a) possesses a different helical symmetry parametrized by 1/*r* on each magnetic surface $r=\mathrm{constant}$.

Similarly, the flux function $\Psi$ defined by Eq. (15c) is not invariant under continuous Euclidean isometries. Indeed, the equation

21 $$\begin{aligned} \mathfrak {L}_{\varvec{\xi }}\Psi =\varvec{\xi }\cdot \nabla \Psi =0,~~~~\varvec{\xi }=\varvec{a}+\varvec{b}\times \varvec{x}, \end{aligned}$$

does not have solution for any nontrivial choice of $\varvec{a},\varvec{b}\in \mathbb {R}^3$. This can be verified easily for $\left|{m}\right|>1$. Indeed, in this case it is sufficient to evaluate $\varvec{\xi }\cdot \nabla \Psi$ over the line $r=r_0$, $z=0$ parametrized by $\varphi$. Here, we have

22 $$\begin{aligned} \begin{aligned} \varvec{\xi }\cdot \nabla \Psi =&-\epsilon \mathcal {E}\sin \left( {m\varphi }\right) \varvec{\xi }\cdot \nabla \left( {z-\epsilon \sin \eta }\right) \\ =&-\epsilon \mathcal {E}\sin \left( {m\varphi }\right) \left[ a_z-\frac{\epsilon a_z}{r_0}\cos \left( {m\varphi }\right) +r_0b_x\sin \varphi -r_0b_y\cos \varphi -\epsilon \left( {b_x-\frac{ma_x}{r_0}}\right) \sin \varphi \cos \left( {m\varphi }\right) \right. \\ {}&\left. +\epsilon \left( {b_y-\frac{ma_y}{r_0}}\right) \cos \varphi \cos \left( {m\varphi }\right) -\epsilon m b_z\cos \left( {m\varphi }\right) \right] . \end{aligned} \end{aligned}$$

This quantity identically vanishes provided that $a_x=a_y=a_z=b_x=b_y=b_z=0$.

## Properties of the constructed solutions

**Figure 3:**
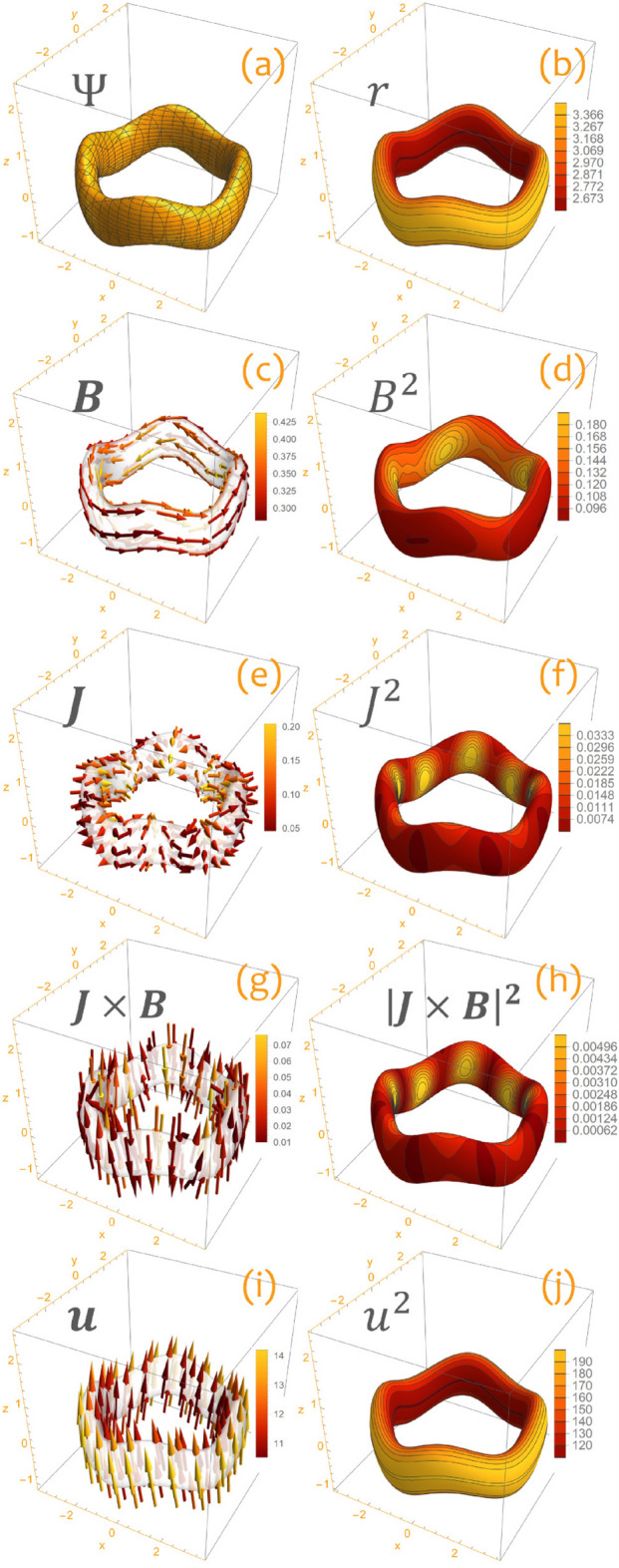
The quasisymmetric configuration (15) for $r_0=3$, $\epsilon =0.2$, $m=4$ and $\mathcal {E}=0.7$. (**a**) Flux surface $\Psi =0.1$. (**b**) Levels sets of *r* on the flux surface $\Psi =0.1$. These contours correspond to magnetic field lines. (**c**), (**d**), (**e**), (**f**), (**g**), (**h**), (**i**), (**j**): plots of the magnetic field $\varvec{B}$, the modulus $B^2$, the electric current $\varvec{J}$, the modulus $J^2$, the Lorentz force $\varvec{J}\times \varvec{B}$, the modulus $\left|{\varvec{J}\times \varvec{B}}\right|^2$, the quasisymmetry $\varvec{u}$, and the modulus $u^2$ on the flux surface $\Psi =0.1$. Figure created using Wolfram Mathematica 12.2 (www.wolfram.com/mathematica).

Let us examine the properties of the quasisymmetric configuration (15). First, observe that level sets of (15c) define toroidal surfaces (see Fig. 3a), implying that the magnetic field (15a) has nested flux surfaces. Next, note that the function $\zeta$ such that $\varvec{B}\times \varvec{u}=\nabla \zeta$ is proportional to the radial coordinate, i.e. $\zeta =mr$. This function is associated with the conserved momentum $\bar{p}$ generated by the quasisymmetry. In particular, we have^5^

23 $$\begin{aligned} \bar{p}=-\frac{1}{\epsilon _\mathrm{gc}}\zeta +v_{\parallel }\frac{\varvec{u}\cdot \varvec{B}}{B}. \end{aligned}$$

Here, $v_{\parallel }$ denotes the component of the velocity of a charged particle along the magnetic field $\varvec{B}$ while $\epsilon _{\mathrm{gc}}\sim \rho /L$ is a small parameter associated with guiding center ordering, $\rho$ the gyroradius, and *L* a characteristic length scale for the magnetic field. It follows that charged particles moving in the magnetic field (15a) will approximately preserve their radial position since $\bar{p}\approx -\frac{m}{\epsilon _{\mathrm{gc}}}r$. This property works in favor of good confinement, although it cannot prevent particles from drifting in the vertical direction. The situation is thus analogous to the case of an axially symmetric vacuum magnetic field $\varvec{B}_{0}=\nabla \varphi$. Level sets of $\zeta =mr$ on a flux surface (15c) are shown in Fig. 3b. These contours correspond to magnetic field lines because the magnetic field (15a) is such that $\varvec{B}\cdot \nabla \Psi =\varvec{B}\cdot \nabla r=0$, and field lines are solutions of the ordinary differential equation $\dot{\varvec{x}}=\varvec{B}$. In particular, observe that magnetic field lines are not twisted (the rotational transform is zero), and are given by the intersections of the surfaces $\Psi =\mathrm{constant}$ and $r=\mathrm{constant}$, implying that their projection on the $\left( {x,y}\right)$ plane is a circle. Plots of the magnetic field (15a) and its modulus $B^2$ are given in Fig. 3c, d. It is also worth noticing that the magnetic field (15a) is not a vacuum field. Indeed, it has a non-vanishing current $\varvec{J}=\nabla \times \varvec{B}$ given by

24 $$\begin{aligned} \varvec{J}=\frac{\epsilon }{r^3}\left[ -\left( {1+m^2}\right) \sin \eta \nabla r+m\left( {2r\cos \eta -z\sin \eta }\right) \nabla \varphi +\left( {\cos \eta -\frac{z}{r}\sin \eta }\right) \nabla z\right] . \end{aligned}$$

Figures 3e, f show plots of the current field $\varvec{J}$ and the corresponding modulus $J^2$. The Lorentz force $\varvec{J}\times \varvec{B}$ can be evaluated to be

25 $$\begin{aligned} \begin{aligned} \varvec{J}\times \varvec{B}=&\frac{\epsilon }{r^4}\left\{ \left[ \left( {\cos \eta -\frac{z}{r}\sin \eta }\right) \left( {\epsilon \left( {1+m^2}\right) \frac{\cos \eta }{r}-1}\right) +\epsilon m^2\frac{\cos ^2\eta }{r}\right] \nabla r\right. \\ {}&\left. +\epsilon m\left( {1+m^2}\right) \sin \eta \cos \eta \nabla \varphi -\left( {1+m^2}\right) \sin \eta \left( {1-\epsilon \frac{\cos \eta }{r}}\right) \nabla z \right\} . \end{aligned} \end{aligned}$$

It is not difficult to verify that the right-hand side of this equation cannot be written as the gradient of a pressure field $\nabla P$. Hence, the quasisymmetric magnetic field (15a) does not represent an equilibrium of ideal magnetohydrodynamics. Nevertheless, it can be regarded as an equilibrium of anistropic magnetohydrodynamics $\varvec{J}\times \varvec{B}=\nabla \cdot \Pi$ provided that the components $P_{\perp },P_{\parallel }$ of the pressure tensor $\Pi ^{ij}=P_{\perp }\delta ^{ij}+\left( {P_{\parallel }-P_{\perp }}\right) B^iB^j/B^2$ are appropriately chosen. Indeed, it is sufficient to set $P_{\perp }=\left( {P_0-B^2}\right) /2$ and $P_{\parallel }=\left( {P_0+B^2}\right) /2$ with $P_0$ a real constant (on this point, see^26^). Plots of the Lorentz force $\varvec{J}\times \varvec{B}$ and its modulus $\left|{\varvec{J}\times \varvec{B}}\right|^2$ are given in Figs. 3g, h. Next, observe that the quasisymmetry $\varvec{u}$ given by Eq. (15b) is not tangential to the toroidal flux surfaces $\Psi$ defined in (15c). Indeed,

26 $$\begin{aligned} \varvec{u}\cdot \nabla \Psi =m\mathcal {E}\left( {z-\epsilon \sin \eta }\right) r. \end{aligned}$$

Plots of the quasisymmetry $\varvec{u}$ and its modulus $u^2$ can be found in Fig. 3i, j.

Finally, let us consider how the quasisymmetry of the configuration (15) compares with the usual understanding that the modulus of a quasisymmetric magnetic field depends on a flux function $\Psi _b$ and a linear combination of toroidal angle $\varphi _b$ and poloidal angle $\vartheta _b$, i.e. $B^2\left( {\Psi _b,M\vartheta _b-N\varphi _b}\right)$ with *M*, *N* integers. When $B^2=B^2\left( {\Psi _b,M\vartheta _b-N\varphi _b}\right)$, on each flux surface the contours of the modulus $B^2$ in the $\left( {\varphi _b,\vartheta _b}\right)$ plane form straight lines. For the quasisymmetric magnetic field (15a) we have $B^2=B^2\left( {r,m\varphi +z/r}\right)$. Hence, the correspondence with the usual setting can be obtained by the identification $\Psi _b\rightarrow r$, $\varphi _b\rightarrow \varphi$, and $\vartheta _b\rightarrow z/r$. This correspondence can be made more rigorous by recalling that the property $B^2=B^2\left( {\Psi _b,M\vartheta _b-N\varphi _b}\right)$ arises from writing the triple vector product formulation of quasisymmetry, $\nabla \Psi _b\times \nabla B\cdot \nabla \left( {\varvec{B}\cdot \nabla B}\right) =0$, through Boozer coordinates $\left( {\Psi _b,\varphi _b,\vartheta _b}\right)$. In these coordinates the magnetic field has expression $\varvec{B}=B_{\Psi _b}\left( {\Psi _b,\varphi _b,\vartheta _b}\right) \nabla \Psi _b+B_{\varphi _b}\left( {\Psi _b}\right) \nabla \varphi _b+B_{\vartheta _b}\left( {\Psi _b}\right) \nabla \vartheta _b$, which implies $\varvec{J}\cdot \nabla \Psi _b=0$. This is a property satisfied by magnetohydrodynamic equilibria with isotropic pressure. However, as discussed above the solution (15a) does not belong to the class of magnetohydrodynamic equilibria with isotropic pressure. Therefore, the existence of Boozer coordinates is nontrivial. Nevertheless, for the solution (15a) it is possible to identify generalized Boozer coordinates $\left( {\Psi _{gb},\varphi _{gb},\vartheta _{gb}}\right) =\left( {r,\eta /r,-z/r}\right)$ with the property that the Jacobian $\mathcal {J}=\nabla \Psi _{gb}\cdot \nabla \varphi _{gb}\times \nabla \vartheta _{gb}=-m/r^3$ is a function of the flux function $\Psi _{gb}=r$ and the quasisymmetry is expressed by the condition $\partial B/\partial \vartheta _{gb}=0$ or $B^2=B^2\left( {r,m\varphi +z/r}\right)$ (on this point, see^6^).

**Figure 4:**
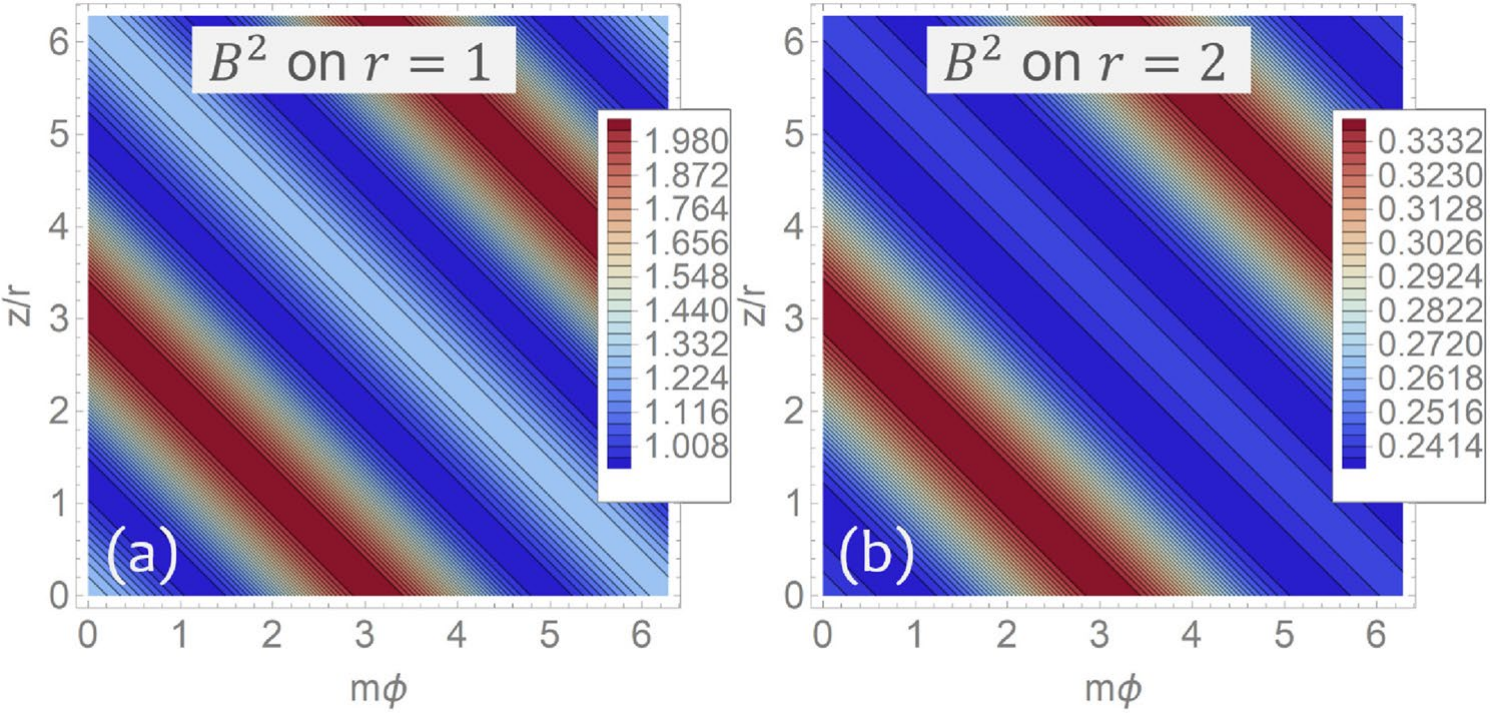
Modulus $B^2\left( {r,m\varphi +z/r}\right)$ of the quasisymmetric magnetic field (15a) for $\epsilon =0.2$ and $m=4$ as seen in the $\left( {m\varphi ,z/r}\right)$ plane for different values of the radial coordinate *r*. (**a**) Plot on the level set $r=1$. (**b**) Plot on the level set $r=2$. Observe how contours of $B^2$ form straight lines. Figure created using Wolfram Mathematica 12.2 (www.wolfram.com/mathematica).

**Figure 5:**
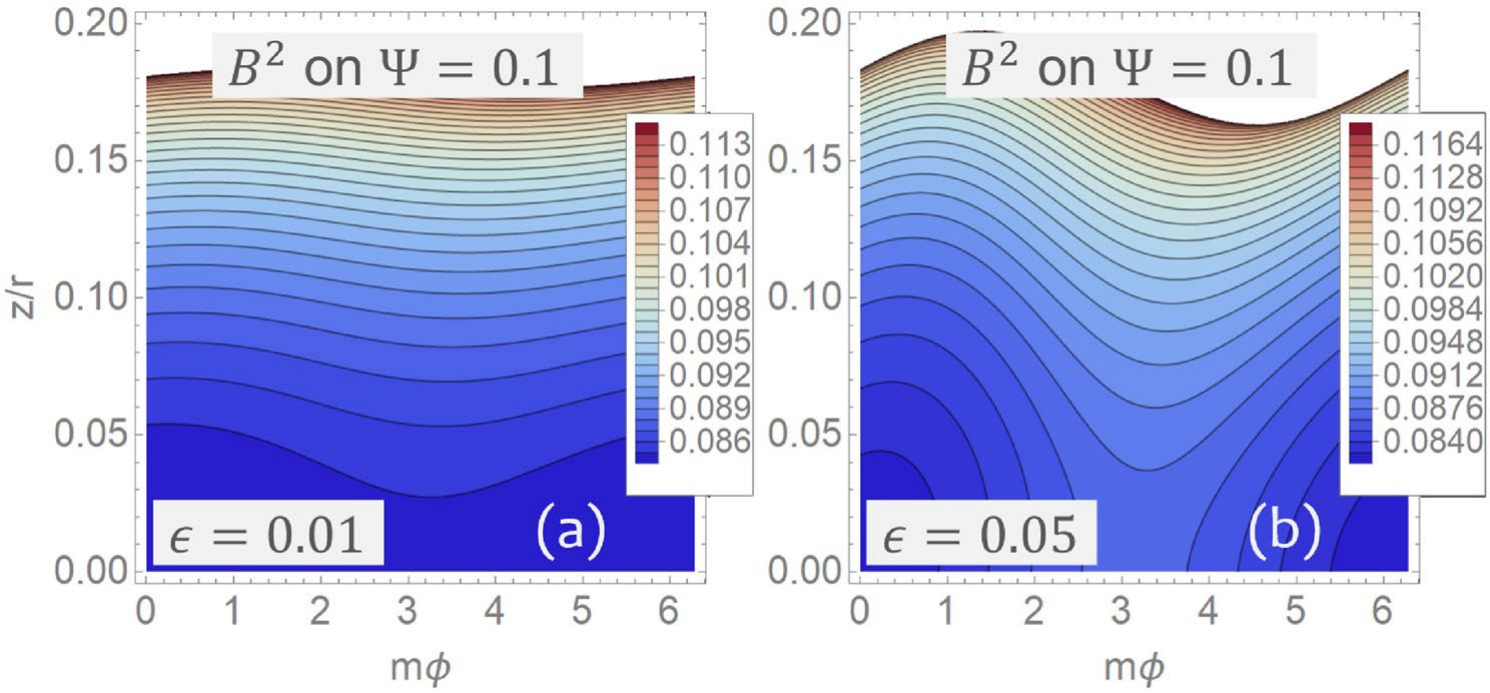
Modulus $B^2\left( {r\left( {\Psi ,z/r,\eta }\right) ,\eta }\right)$ with $\eta =m\varphi +z/r$ of the quasisymmetric magnetic field (15a) for $r_0=3$, $m=4$ and $\mathcal {E}=0.7$ as seen in the $\left( {m\varphi ,z/r}\right)$ plane corresponding to $\Psi =0.1$. (**a**) The case $\epsilon =0.01$. (**b**) The case $\epsilon =0.05$. Notice that white regions in the plot reflect the fact that for given values of $\Psi$ and $\varphi$ the range of *z* is bounded. Figure created using Wolfram Mathematica 12.2 (www.wolfram.com/mathematica).

Figure 4 shows how the contours of the quasisymmetric magnetic field (15a) form straight lines in the $\left( {m\varphi ,z/r}\right)$ plane. Next, it is useful to determine how much the contours of $B^2$ depart from straight lines on each flux surface $\Psi$. To this end, observe that Eq. (15c) can be inverted to obtain $r\left( {\Psi ,z/r,\eta }\right)$ with $\eta =m\varphi +z/r$ so that the modulus (17) can be written in the form $B^2=B^2\left( {r\left( {\Psi ,z/r,\eta }\right) ,\eta }\right)$. Figure 5 shows contours of $B^2$ on the plane $\left( {m\varphi ,z/r}\right)$ for a fixed value of $\Psi$ and different choices of the parameter $\epsilon$ controlling the degree of asymmetry of the solution. In particular, notice how the solution (15) approaches axial symmetry for smaller values of $\epsilon$.

## Concluding remarks

In conclusion, we have demonstrated the existence of weakly quasisymmetric magnetic fields in toroidal volumes by constructing explicit examples (14) through the method of Clebsch parametrization. The obtained configurations are solutions of system (1) with the following properties. In the optimized toroidal domain $\Omega$, the magnetic field $\varvec{B}$ is smooth and equipped with nested flux surfaces $\Psi$. Both $\varvec{B}$ and $\Psi$ do not exhibit continuous Euclidean isometries, i.e. invariance under an appropriate combination of translations and rotations. The magnetic field $\varvec{B}$ has vanishing rotational transform, while the quasisymmetry $\varvec{u}$ is not tangential to contours of the flux function $\Psi$ defined in (14c), but lies on surfaces of constant radius *r*. In particular, $\varvec{B}\times \varvec{u}=m\nabla r$ with *m* an integer while $B^2=B^2\left( {r,m\varphi +z/r}\right)$ in the example (15). The conserved momentum arising from the quasisymmetry is given by (23), which is approximately the radial position of a charged particle. The magnetic field $\varvec{B}$ is not a vacuum field since a current $\varvec{J}=\nabla \times \varvec{B}\ne \varvec{0}$ is present. The obtained quasisymmetric magnetic fields (14a) can be regarded as solutions of anisotropic magnetohydrodynamics if the component of the pressure tensor are appropriately chosen^26^.

In addition to providing mathematical proof of existence of solutions to system (1) with the properties described above, this work offers an alternative theoretical framework for the numerical and experimental efforts devoted to modern stellarator design, and possibly paves the way to the development of semi-analytical schemes aimed at the optimization of confining magnetic fields. The next goal of the present theory would be to further improve the obtained results by ascertaining the existence of vacuum solutions $\nabla \times \varvec{B}=\varvec{0}$ of system (1) such that the modulus of the magnetic field can be written as a function of the flux function and a linear combination of toroidal and poloidal angles, $B^2=B^2\left( {\Psi ,M\vartheta -N\varphi }\right)$, and in particular to establish the existence of vacuum quasisymmetric configurations with the field line twist required to effectively trap charged particles.

## Data Availability

The datasets generated during and/or analysed during the current study are available from the corresponding author on reasonable request.
